# Caracterización clínica, sociodemográfica y determinación del impacto en la calidad de vida de pacientes con dermatitis atópica de la ciudad de Medellín y su área metropolitana

**DOI:** 10.7705/biomedica.5978

**Published:** 2021-12-15

**Authors:** Miguel Mateo Cuervo, Gloria Sanclemente, Lina Marcela Barrera

**Affiliations:** 1 Facultad de Medicina, Sección Dermatología, Universidad de Antioquia, Medellín, Colombia Universidad de Antioquia Facultad de Medicina Universidad de Antioquia Medellín Colombia; 2 Grupo de Investigación Dermatológica, Universidad de Antioquia, Medellín, Colombia Universidad de Antioquia Grupo de Investigación Dermatológica Universidad de Antioquia Medellín Colombia

**Keywords:** dermatitis atópica/epidemiología, calidad de vida, sueño, depresión, Dermatitis, atopic/epidemiology, quality of life, sleeping, depression

## Abstract

**Introducción.:**

La dermatitis atópica es una enfermedad cutánea crónica e intermitente muy frecuente, con un impacto clínico evidente en la calidad de vida de los pacientes. No hay estudios locales que describan las características generales de esta condición en nuestra población.

**Objetivo.:**

Evaluar las características sociodemográficas, clínicas y de calidad de vida en pacientes con dermatitis atópica residentes en Medellín y su área metropolitana.

**Materiales y métodos.:**

Se hizo un estudio transversal descriptivo de pacientes que consultaron en algunos centros de referencia de la ciudad de Medellín, a quienes se les hizo una encuesta sobre variables sociodemográficas y clínicas. Además, se evaluó la seriedad clínica de la enfermedad y su impacto en la calidad de vida.

**Resultados.:**

Se incluyeron 113 pacientes, de los cuales 36,3 % manifestó tener algún trastorno del sueño, 38,1 % reportó déficit de atención y 44,2 % informó haber sufrido asma en la infancia. Se registró un puntaje promedio de 6,9 en el índice EASI (*Eczema Area and Severity Index*) y de 32,4 en el SCORAD (*Scoring Atopic Dermatitis*), es decir, el compromiso clínico era de leve a moderado. Con el cuestionario Skindex-29, se encontraron promedios de 37,7, 25,09 y 16,9 para los dominios sintomático, emocional y funcional, respectivamente, con un promedio total de 24,78. En el cuestionario *EuroQol 5 Dimensions* (EQ-5D), el componente más importante fue la presencia de dolor o malestar (17,6 %) y de ansiedad o depresión (12,1 %).

**Conclusión.:**

Los resultados obtenidos confirmaron que la población analizada con dermatitis atópica presentaba características similares a las reportadas en otras partes del mundo, con un impacto neuropsiquiátrico y emocional en aspectos como el sueño.

## Abstract (en)

**Introduction:**

Atopic dermatitis is a cutaneous, inflammatory, chronic disease, very frequent in the world and it imposes a high clinical and quality of life impact in patients. To date there are no local studies describing the general features in our population.

**Objective:**

To evaluate the socio demographic, clinical and life quality features in patients with atopic dermatitis from Medellín city and its metropolitan area.

**Materials and methods:**

cross sectional, descriptive study, that included patients who attended some teaching dermatological clinics. Data collection included sociodemographic and clinical features in addition to clinical issues and quality of life measurements.

**Results:**

A total of 13 patients were included. 36,3% manifested a sleeping disturbance, 38,1% attention deficit; 44,2% reported asthma in the childhood. EASI and SCORAD scores were 6,9 and 32,4, respectively, which indicates a mild-moderate compromise. Regarding quality of Life, Skindex-29 showed average scores for the symptomatic, emotional and functional dominions of 37,7, 25,09 and 16,9, respectively, and a total score of 24,78. In EQ-5D scores, the most altered component was the presence of pain or discomfort (17,6%) and the presence of anxiety or depression (12,1%), which enhances the symptomatic and emotional impact of the disease.

**Conclusion:**

These results confirm that patients with atopic dermatitis present similar features to those reported in other studies from different countries, highlighting the burden of the disease in adults and its impact in neuropsychiatric and emotional domains.,

La dermatitis atópica es una enfermedad inflamatoria crónica que suele aparecer en la infancia temprana, aunque también puede presentarse en otras edades. Afecta a ambos sexos y a todas las razas. En su etiología intervienen factores genéticos, ambientales e infecciosos que provocan una reacción inflamatoria en la piel, con ruptura de la barrera cutánea y aparición de las lesiones clínicas características ^1^. Su fisiopatología es compleja y aún no está completamente dilucidada. Hoy se conocen múltiples vías de señalización adicionales al eje Th1-Th2, entre ellas, la producción de IL-3, IL-4 e inmunoglobulina E (IgE), la intervención de linfocitos con linaje Th17, la producción de otras citocinas como la IL-17, IL-18 y la IL-22, y algunas mutaciones genéticas que provocan anormalidades en proteínas de barrera de la epidermis ^2^^-^^5^.

La enfermedad se ha convertido rápidamente en un problema de salud pública debido a su gran prevalencia, especialmente en países industrializados, y a los altos costos y la carga económica que implica ^2^, pues suele iniciarse en los dos primeros años de vida. Se ha descrito, además, una curva ascendente de la prevalencia, la cual pasó del 8 % de afectados en 1997 al 12 % en 2010 ^6^. Los datos provenientes de los diversos países varían mucho, según el sitio de los estudios. Por ejemplo, el llevado a cabo entre 1999 y 2004 mediante el cuestionario del ISAAC (*International Study of Asthma and Allergies in Childhood*), incluyó 385.853 pacientes entre los 6 y 7 años de edad provenientes de 60 países de todos los continentes; en dicho grupo se reportó una prevalencia de la dermatitis atópica de 0,2 a 24,6 %, y Colombia figuró entre los países con mayores prevalencias generales (>15 %), y en el grupo de 13 a 14 años de edad (alrededor de 600.000 individuos), con 24,6 % ^7^.

En 2004, Dennis, *et al*., evaluaron la prevalencia de enfermedades alérgicas en seis ciudades colombianas y encontraron que, de un total de 6.507 pacientes, el 3,9 % presentaba dermatitis atópica y un tercio de ellos tenía síntomas graves; en Medellín se reportó una prevalencia de 4 % (39 pacientes) ^8^. En un nuevo estudio de los mismos autores entre 2009 y 2010, se encontró una prevalencia estimada de dermatitis atópica de 13,89 % en una población de 5.780 individuos, y Medellín registró el valor más alto: 13,36 % (176 pacientes). Además, se encontró que el 13 % de los individuos manifestaba despertarse una o más noches a la semana por síntomas relacionados con la enfermedad ^9^. En 2012, se evaluaron niños entre los 5 y 14 años en Medellín para establecer la prevalencia de enfermedades alérgicas y su relación con el trastorno de déficit de atención con hiperactividad; se encontró que, de 113 pacientes encuestados, el 8,8 % presentaba dermatitis atópica, condición que, de todas las evaluadas, se asoció significativamente con el insomnio (OR=4,23), en tanto que el déficit de atención con hiperactividad registró una prevalencia similar a la de la población general ^10^^,^^11^.

Lo usual en la dermatitis atópica es que se presenten brotes y, en general, los pacientes comparten características como el eccema y el prurito y la tendencia a tener la piel xerótica. Asimismo, algunos individuos pueden presentar otros signos, denominados estigmas de atopia, como piel pálida, presencia de doble pliegue palpebral, e hiperlinealidad palmar, entre otras ^12^^-^^14^. Los pacientes tienen exacerbaciones y remisiones que suelen durar más de seis semanas; durante las exacerbaciones, las lesiones se generalizan y se presentan sobreinfecciones, incluso, en algunos pacientes aparecen formas eritrodérmicas que se extienden a más del 70 al 90 % de la superficie corporal ^15^. Para su diagnóstico, se utilizan criterios clínicos que las diversas organizaciones dermatológicas mundiales han venido modificando a través de los años ^16^^,^^17^. En general, los signos esenciales son la presencia de eccema o prurito a lo largo de la vida y la presencia de exacerbaciones ^18^.

Se sabe que la dermatitis atópica provoca alteraciones clínicas que afectan la calidad de vida del paciente y de su familia, por lo que se han diseñado diferentes escalas para medir tal impacto ^15^^,^^19^. Con el EASI (*Eczema Area and Severity Index*), índice publicado en 1998 ^20^, se evalúa la extensión de la enfermedad en cuatro regiones definidas del cuerpo y se determina la gravedad según cuatro signos clínicos: eritema, induración o palpación, excoriación y liquenificación; a cada signo se le da un valor en una escala que varía entre 0 si el signo está ausente y 3 si es grave, y se le da un peso a dichos signos de acuerdo con la extensión del área anatómica afectada. La extensión se puntúa de 0 a 6: 0 corresponde a un compromiso del 0 % y 6, a uno del 90 a 100 %. Finalmente, se obtiene un puntaje que va de 0 a 72. El compromiso se considera leve con un puntaje entre 1,1 y 7, moderado, entre 7,1 y 21, grave, entre 21,1 y 50, y muy grave, entre 50,1 y 72 ^21^.

El SCORAD (*Scoring Atopic Dermatitis*), por su parte, es una de las herramientas más ampliamente utilizadas. Se publicó en 1993 y permite evaluar tres aspectos de la enfermedad: extensión, características clínicas y síntomas. La extensión se determina calculando la superficie corporal que se encuentra afectada. Las características clínicas, el eritema, las pápulas o el edema, la exudación o costras, la excoriación, la liquenificación y la xerosis, a cada una de las cuales se le da un puntaje de 0 a 3 (0 si la característica está ausente y 3 si es grave). Los síntomas de prurito y alteración del sueño son puntuados por la persona en una escala visual análoga. En general, el compromiso se considera leve cuando el puntaje es menor de 15, moderado si está entre 15 y 40, y grave si es mayor de 40 ^22^.

Para la evaluación de la calidad de vida se emplearon las escalas Skindex-29 y *EuroQol 5 Dimensions* (EQ-5D), las cuales fueron diligenciadas por los mismos pacientes. El Skindex-29 es un cuestionario diligenciado por el paciente que incluye 29 ítems distribuidos en tres dominios: sintomático, emocional y funcional. En cada pregunta debe responderse con la frecuencia de cada situación: nunca, raramente, algunas veces, frecuentemente y todo el tiempo. Los puntos de corte para cada dominio se establecieron así: leve, 20 a 30; moderado, entre 30 y 40, y grave, de 40 o más ^23^. La EQ- 5D es una herramienta para evaluar la calidad de vida de las personas con enfermedades dermatológicas, según la movilidad, el cuidado personal, las actividades cotidianas, la presencia o ausencia de dolor o malestar, y de ansiedad o depresión. Es un cuestionario que diligencia el mismo paciente, adjudicando un valor entre 1 y 3 a cada variable, siendo 1 la ausencia de alteración, 2 la presencia de afectación leve o moderada y 3 el compromiso serio; además, los pacientes deben elegir un puntaje que va de 0 a 100 como promedio de su estado de salud en el día de la evaluación, siendo 0 el peor estado de salud y 100 el mejor ^24^.

Actualmente, no hay un tratamiento único para la dermatitis atópica, aunque sí múltiples terapias para diferentes etapas de la enfermedad que, combinadas con la educación de los pacientes, constituyen el plan de intervención ideal ^25^^,^^26^. La condición por sí misma es un problema para los pacientes, pues uno de los síntomas más importantes es el prurito, el cual puede ser muy difícil de controlar e impacta negativamente su calidad de vida que, en conjunto con otros síntomas, aumenta el ausentismo laboral, así como la asistencia física al lugar de trabajo, pero con un desempeño inadecuado de las tareas asignadas. Asimismo, la enfermedad afecta a los cuidadores directos, pues los pacientes suelen ser niños, y tiene una gran repercusión sobre el sistema de salud por la cantidad de consultas que los pacientes requieren y la complejidad del tratamiento ^13^^,^^27^^-^^29^.

Hasta la fecha, se desconoce si en la población del área metropolitana de Medellín la enfermedad guarda alguna relación con los factores externos que se han señalado en diversos estudios en asociación con su origen y desarrollo, así como con otras enfermedades. Se reconoce su impacto en los pacientes, pero es importante saber si en la población local se manifiesta de la misma manera para que, tanto los dermatólogos como otros profesionales de la salud (médicos generales, pediatras, alergólogos, internistas), tengan los elementos para reconocer la enfermedad, su gravedad y las comorbilidades, y puedan darle un manejo integral a los pacientes.

En este contexto se inscribe la pregunta que guió este estudio: ¿cuál es la distribución clínica y sociodemográfica, y el impacto en la calidad de vida de los pacientes con dermatitis atópica que consultan en Medellín y su área metropolitana?

## Materiales y métodos

### Población de estudio

Se hizo un estudio de tipo descriptivo en todos los pacientes con dermatitis atópica que de manera consecutiva asistieron al servicio de dermatología de los siguientes centros asistenciales: IPS Universitaria, Hospital Universitario San Vicente Fundación, Clínica Bolivariana, Universidad CES y algunos consultorios dermatológicos particulares de la ciudad y su área metropolitana en el periodo comprendido entre junio de 2018 y marzo de 2020.

Se incluyeron los pacientes colombianos con más de 7 años de edad y diagnóstico clínico de dermatitis atópica emitido por un dermatólogo, que aceptaron participar en el estudio diligenciando el formato de consentimiento informado y autorizando el acceso a sus registros clínicos y sus datos personales; en el caso de los niños entre 7 y 12 años, se debía obtener, además,el asentimiento informado; se excluyeron los pacientes que no cumplieran dichos requisitos. El Skindex-29 se aplicó a todos los pacientes mayores de 16 años y el EQ5D solo a los adultos, edades para las que se han validado estas escalas.

### Tamaño de muestra y muestreo

El muestreo fue a conveniencia y el tamaño de la muestra se calculó con base en una frecuencia en la población del 11 % ^30^, con un intervalo de confianza del 90 % para una población finita mayor de 1’000.000 con un efecto de diseño (para encuestas en el grupo-EDF) de 1, según la siguiente fórmula (https://www.openepi.com/SampleSize/SSPropor.htm):

Tamaño de la muestra n = [EDF*Np(1-p)] / [(d^2^/Z^2^_1-α/_2*(N-1)+p*(1-p)], lo que arroj**ó** un n de 106 pacientes.

### Recolección de datos

Se invitó a participar en el estudio a todos los pacientes atendidos en los centros asistenciales seleccionados y se hizo su registro inicial con nombre, documento de identificación y teléfono de contacto; posteriormente, un residente de dermatología de la Universidad de Antioquia se comunicó con cada uno para establecer una cita presencial en la IPS Universitaria o en el Hospital Universitario San Vicente Fundación, donde se diligenció el formato de consentimiento informado y se hicieron las encuestas del estudio.

Asimismo, durante la recolección de datos se organizaron jornadas en la IPS Universitaria dirigidas a pacientes con dermatitis atópica que fueron difundidas por medios físicos y digitales, y en las cuales se hizo un acercamiento inicial para explicar los objetivos de la investigación y remitir a consulta especializada a quienes lo requirieran; los pacientes que aceptaban participar en el estudio fueron encuestados inmediatamente. En dichas jornadas también se dictaron charlas a cargo de residentes de dermatología de la Universidad de Antioquia para informar sobre los aspectos generales de la enfermedad, los cuidados que debían tener los pacientes y el propósito de la investigación.

A todos los pacientes que cumplían con los criterios de inclusión se les aplicó una encuesta diligenciada por residentes de dermatología de la Universidad de Antioquia para recoger los datos sociodemográficos de edad, fecha de nacimiento, sexo, lugar de origen (ciudad y departamento), estado civil, ocupación desempeñada en los últimos cinco años, escolaridad, estrato socioeconómico y aseguramiento en salud, así como información sobre los ingresos mensuales promedio, la dependencia económica (en caso de haberla) y el dinero invertido mensualmente en la enfermedad.

Para evaluar las características clínicas y comorbilidades, la encuesta incluía los datos de peso (kg), estatura (cm), consumo de cigarrillo, alcohol y sustancias psicoactivas, antecedentes personales psiquiátricos, trastornos del sueño, historia personal y familiar de atopia, otras comorbilidades y presencia de estigmas de atopia. La gravedad clínica se midió con base en el porcentaje de superficie corporal comprometida mediante los instrumentos EASI y SCORAD.

### Análisis estadístico

Los datos obtenidos con las encuestas se transcribieron e ingresaron a una base de datos de Microsoft Excel® 2013 y de Access® 2013. Para la descripción de las características de la muestra, se hizo un análisis univariado de las variables para conocer sus frecuencias. Las variables continuas se presentaron utilizando medias y desviación estándar, o medianas y rango intercuartil. Las variables ordinales y categóricas se expresaron en frecuencias absolutas, y las relativas en porcentajes. Para el análisis estadístico de los datos se utilizó el programa SPSS®, versión 26.

### Consideraciones éticas

El estudio se ajustó a las normas de investigación en seres humanos estipuladas en la Resolución N° 8430 de 1993 del Ministerio de Salud de Colombia y en la Declaración de Helsinki de 2013. El estudio supuso un riesgo mínimo para los participantes y se incluyeron únicamente los pacientes que manifestaran su deseo de participar y diligenciaran el consentimiento informado. Los niños entre 7 y 12 años debían otorgar, además, el asentimiento informado. En la base de datos se asignaron códigos numéricos para mantener la confidencialidad de la información.

El estudio fue aprobado por el comité de ética de la IPS Universitaria, el Hospital San Vicente Fundación y el Instituto de Investigaciones Médicas de la Facultad de Medicina de la Universidad de Antioquia. No hubo ningún conflicto de intereses económicos personales o que involucraran a terceros en la realización de esta investigación.

## Resultados

El estudio incluyó a 113 pacientes atendidos en el periodo comprendido entre junio de 2018 y marzo de 2020, cuyas características sociodemográficas se presentan en el cuadro 1. El promedio de edad de los pacientes evaluados fue de 27,7 años (rango de 7 a 68 años); 23 pacientes eran menores de 18 años. La distribución por sexo fue muy similar: 57 mujeres (50,4 %) y 56 hombres (49,6 %). En cuanto al estado civil, la mayoría eran solteros (71, 62,8 %); el 33,6 % era profesional universitario, el 31,9 %, bachiller y el 15,9 %, técnicos. El 70,8 % pertenecía al régimen contributivo del sistema de seguridad socia y el 18,6 % al subsidiado. En el cuadro 2 se presenta la caracterización económica: el 70,8 % de los pacientes dependía económicamente de alguien más, y 48 de ellos (42,5 %) no tenía ingresos económicos.

**Cuadro 1:** Características sociodemográficas de la población de estudio

Sexo	Mujeres, 50,4 % (57)
	Hombres, 49,6 % (56)
Edad promedio	27,7 años (7-68 años)* (DE=17,14)
Estado civil	Soltero, 62,8 % (71)
	Unión libre, 30,1% (34)
	Casado, 5,3 % (6)
	Viudo, 0,9 % (1)
Escolaridad	Ninguna, 3,5 % (4)
	Primaria, 6,2 % (7)
	Secundaria, 31,9 % (36)
	Técnico, 15,9 % (18)
	Profesional, 33,6 % (38)
	Posgrado, 8,8 % (10)
Aseguramiento en salud	Ninguno, 0,9 % (1)
	Contributivo, 70,8 % (80)
	Subsidiado, 18,6 % (21)
	Particular, 5,3 % (6)
	Régimen especial, 2,7 % (3)
	SISBEN, 1,8 % (2)
Total	N = 113

DE: desviación estándar

* 23 (20,3 %) pacientes eran menores de 18 años.

**Cuadro 2:** Características económicas de la población de estudio

Dependencia económica	Sí, 70,8 % (80)
	No, 29,2 % (33)
Ingresos mensuales*	Sin ingreso, 42,5 % (48)
	$ 0 a $ 500.000, 12,4 % (14)
	$ 500.000 a $ 1’000.000 12,4 % (14)
	$ 1’000.000 a $ 2’000.000, 15,9 % (18)
	$2.000.000 a $ 5.000.000, 11,5 % (13)
	$2.000.000 a $ 5.000.000, 11,5 % (13)
	$ 5’000.000 a $ 10’000.000, 2,7 % (3)
	>$ 10’000.000, 1,8 % (2)
Inversión mensual en la enfermedad**	$ 0 a $ 100.000, 43,3 % (49)
	$ 100.000 a $ 200.000, 23 % (26)
	$ 200.000 a $ 500.000 23,9 % (27)
	$ 500.000 a $ 1’000.000 4,4 % (5)
Total	N = 106

*Uno de los pacientes no reportó ingreso mensual

** Seis pacientes no reportaron su gasto mensual por la enfermedad

### Comorbilidades personales y factores de riesgo familiares

Las comorbilidades y antecedentes familiares se presentan en el cuadro 3. El 14,2 % de los pacientes refirió consumo actual de cigarrillo, el 41,6 % de alcohol y el 9,7 % de sustancias psicoactivas. Por otro lado, 41 (36,3 %) pacientes manifestaron tener algún trastorno del sueño, 38,1 % expresó tener déficit de atención y el 0,9 %, haber presentado depresión. En cuanto a los antecedentes personales de atopia, 25 (22,1 %) pacientes refirieron presentar eccema desde la infancia, 49 (43,3 %) tenían diagnóstico de dermatitis atópica infantil, 50 (44,2 %) manifestaron haber tenido asma en la infancia y 52 (46 %), rinitis alérgica. El antecedente familiar de dermatitis atópica fue positivo en 93 (82,3 %) pacientes, de asma en 40 (35,3 %) y de rinitis alérgica en 41 (36,2 %). Otras comorbilidades fueron poco frecuentes, siendo la más relevante el asma activa en 7,1 % de los casos, enfermedades de la tiroides en 6,2 %, obesidad y diabetes mellitus no complicada en 1,8 % cada una, y enfermedad pulmonar obstructiva crónica, hígado graso, hipertensión arterial y dislipidemia en 0,9 % de los pacientes cada una.

**Cuadro 3:** Antecedentes y comorbilidades de la población de estudio

Consumo de cigarrillo	Sí, 14,2% (16)
	No, 85,8 % (97)
Consumo de alcohol	Consumo de alcohol
	Consumo de alcohol
Consumo de sustancias psicoactivas	Sí, 9,7 % (11)
	No, 90,3 % (102)
Antecedentes personales psiquiátricos	Déficit de atención, 38,1 % (43)
	Trastornos del sueño, 36,3 % (41)
	Depresión, 0,9 % (1)
Historia personal previa de atopia	Eccema, 22,1 % (25)
	Dermatitis atópica infantil, 44,2 % (50)
	Asma, 44,2 % (50)
	Rinitis alérgica, 46 % (52)
Antecedente familiar de atopia	Dermatitis atópica, 82,3 % (93)
	Asma, 35,3 % (40)
	Rinitis alérgica, 36,2 % (41)
	Alergia alimentaria, 14,1 % (16)
	Alergia a medicamentos, 7,9 % (9)
Otras comorbilidades	Asma activa, 7,1 % (8)
	Enfermedad tiroidea, 6,2 % (7)
	Obesidad, 1,8 % (2)
	Diabetes no complicada, 1,8 % (2)
	EPOC, 0,9 % (1)
	Hígado graso, 0,9 % (1)
	Hipertensión arterial, 0,9 % (1)
	Dislipidemia, 0,9 % (1)

EPOC: enfermedad pulmonar obstructiva crónica

* No se incluye el número total porque algunos pacientes presentaron más de una comorbilidad, antecedente o estigma atópico.

### Características clínicas de la enfermedad y del tratamiento

Las variables clínicas se muestran en el cuadro 4: el promedio del peso fue de 61,3 kg y el de la estatura de 160,9 cm. Según las escalas de clasificación de la gravedad clínica, la intensidad promedio del prurito fue de 5,93; el porcentaje de superficie corporal afectada en el momento de la encuesta fue de 17,14 % en promedio y cuatro pacientes presentaban eritrodermia (>90 % de la superficie corporal afectada). Asimismo, el 97,3 % de los pacientes presentaba algún estigma de atopia, siendo los más comunes la palidez centrofacial (59; 52,2 %) y el doble pliegue palpebral (42; 37,2 %). La más común de las variantes regionales de dermatitis atópica fue el eccema de las manos (14,1 %).

**Cuadro 4:** Características clínicas de la población de estudio

Peso promedio	61,3 kg (DE=17,2)
Estatura promedio	160,9 cm (DE=16,8)
Índice de masa corporal (IMC) promedio	23,68 kg/cm^2^
Porcentaje de superficie corporal	17,14 % (DE=20,5)
afectada en el momento del examen	Eritrodermia, 3,53 % (4)*
Estigmas de atopia	Sí 97,3 % (110) Palidez centrofacial, 52,2 % (59) Doble pliegue palpebral, 37,1 % (42) Hiperpigmentación periocular, 30 % (34) Dermografsmo blanco, 29,2 % (33) Queratosis pilar, 28,3 % (32) Disminución del vello de las cejas, 24,7 % (28) Pitiriasis alba, 24,7 % (28) Hiperlinealidad palmar, 18,5 % (21)
	No, 2,7 % (3)
Variantes de la dermatitis atópica según región del cuerpo	Eccema de las manos, 14,1 % (16)
	Quelitis, 7,9 % (9)
	Eccema auricular, 2,6 % (3)
	Eccema del párpado, 5,3 % (6)
	Dermatitis de cabeza y cuello, 0,9 % (1)
	Prurigo, 1,7 % (2)
	Eccema numular, 3,5% (4)
	Eccema del pezón 3,5% (4)
Tratamiento previo recibido	Fototerapia 35,4 % (40)
	Metotrexate 16,8 % (19)
	Ciclosporina 15,9 % (18)
	Micofenolato mofetil 10,6 % (12)
	Azatioprina 13,3 % (15)
	Producto biológico 13,3 % (15)

DE: desviación estándar

* Eritrodermia definida como porcentaje de afectación de más de 90 %

Se encontró que 105 (92,9 %) pacientes ya habían recibido tratamiento previo para su enfermedad y, de ellos, 40 (35,4 %) habían estado en fototerapia, principalmente con UVB de banda estrecha (31; 27,4 %). En cuanto al tratamiento sistémico, se refirió el uso de metotrexato (16,8 %), ciclosporina (15,9 %), micofenolato de mofetilo (10,6 %), azatioprina (13,3 %) y productos biológicos (13,3 %).

### Escalas de gravedad clínica y de calidad de vida

Los valores de las escalas se presentan en los cuadros 5 y 6. En el EASI, el puntaje promedio fue de 6,9 (con un máximo reportado de 38,9), y en el SCORAD, de 32,4 (con un máximo de 75,5). En el Skindex-29, el promedio para los dominios sintomático, emocional y funcional fue de 37,7, 25,09 y 16,9, respectivamente, con un promedio total de 24,78. En el cuestionario EQ-5D, solo se ingresaron los datos de 91 pacientes, ya que 21 participantes eran menores de edad y un adulto no contestó las preguntas, por lo que se excluyeron del análisis. De los 91 pacientes evaluados, ninguno reportó alteración en la movilidad; el 3,3 % manifestó algún problema con el autocuidado; el 8,8 % presentaba dificultades para la realización de sus actividades diarias; el 17,6 % reportó dolor o malestar (el 16,5 %, algo y el 1,1 %, intenso); por último, el 12,1 % refirió ansiedad o depresión (el 11 %, algo y el 1,1 %, intensa). En cuanto al promedio del estado de salud en el día de la evaluación, el valor fue de 75,01 (rango de 54,48 a 95,54).

**Cuadro 5:** Escalas de gravedad de la enfermedad y calidad de vida

Intensidad promedio del prurito*	EASI	SCORAD	Skindex-29
5,93 (DE=2,9)	6,9 (DE=7,5)	32,4 (DE=19,4)	Dominio sintomático 37,76 (DE=21,6, mediana 35,71)
	Leve, 1,1 a 7	Leve, <15, Moderado, 15 a 40	Dominio emocional 25,09 (DE=22,01, mediana 22,50)
	Moderado, 7,1 a 21 Grave, 21,1 a 50	Grave >40	Dominio funcional 16,94 (DE=18,8, mediana 12,50)
	Muy grave, 50,1 a 72		Total 24,78
			(DE=18,4, mediana 20,69)
			Leve: 20-30
			Moderado: <40
			Grave: ≥40

DE: desviación estándar

* Intensidad del prurito medida por escala visual análoga

**Cuadro 6:** Herramienta EuroQol 5 Dimensions (EQ-5D), N=91, sin dato=22*

Dimensiones	Movilidad	Autocuidado	Actividades diarias	Dolor o malestar Ansiedad o depresión
Nivel	Total	Total	Total	Total
1	91 (100 %)	88 (96,7 %)	83 (91,2 %)	75 (82,4 %) 80 (87,9 %)
2	0 (0 %)	3 (3,3 %)	8 (8,8 %)	15 (16,5 %) 10 (11 %)
3	0	0	0	1 (1,1 %) 1 (1,1 %)

DE: desviación estándar

* 21 pacientes eran menores de edad y hubo un adulto que no contestó las preguntas.

## Discusión

La dermatitis atópica es una enfermedad común de especial relevancia para el dermatólogo, debido a su frecuencia y a su impacto clínico y social. Los datos de grandes estudios internacionales indican que la prevalencia ha venido en aumento en las últimas décadas, con valores de hasta 24 % según el grupo poblacional, pero con promedios que usualmente van del 6 al 12 %. En dichos estudios, Colombia se ha clasificado como un país con una prevalencia alta; por ejemplo, en el estudio ISAAC del 2009 la prevalencia en los diferentes grupos de edad evaluados fue alta (>15 % en pacientes entre 6 y 7 años de edad y hasta 24,6 % en pacientes entre 13 y 14 años). Localmente, también se ha reportado un aumento de la prevalencia, con 4 % en Medellín en el 2004 y 13,36 % en el 2010. Aunque no hay estudios amplios a nivel local que evalúen los grupos de edad más afectados, en otras partes del mundo los más frecuentemente reportados son los niños y los adolescentes.

En este sentido, cabe señalar que en el presente estudio no se incluyeron menores de 6 años. La edad promedio de los participantes fue de 27 años y solo 23 pacientes eran menores de 18 años, lo que resalta la frecuencia de la enfermedad en los adultos, ya que prevalece la idea de que los síntomas mejoran en la adolescencia. Este hallazgo del presente estudio es muy relevante, pues esta es la edad productiva y de inserción laboral. En cuanto al sexo, en múltiples estudios se ha encontrado que la enfermedad es más frecuente en los niños durante la infancia y más en las niñas durante la adolescencia ^31^; en el presente estudio, la relación entre hombres y mujeres fue similar (49,6 % Vs. 50,4 %).

Es bien conocida la relación de la dermatitis atópica con otras condiciones atópicas y alérgicas. En este sentido, hay reportes de asma en hasta el 25 % de los casos y de alergia alimentaria en el 15 %, siendo estos porcentajes incluso más altos en aquellos con síntomas más graves de la enfermedad (hasta el 73 % de los adultos con la forma grave también tiene asma); además, el asma puede ser más frecuente en quienes la enfermedad se inició más tempranamente y en aquellos con bajo nivel de educación y menores ingresos económicos ^32^.

En algunos estudios, se ha reportado que a los tres años de edad el 66 % de los pacientes con dermatitis atópica presenta otra forma de atopia y hasta el 80 % de los niños desarrolla rinitis alérgica o asma, aunque hay niños (especialmente aquellos con los síntomas menos graves) en quienes dichas comorbilidades mejoran cuando llegan a la adultez ^33^.

En el presente estudio, se encontró que el 44,2 % de los pacientes había tenido asma en la infancia y, el 46 %, rinitis alérgica (con un total de 63,7 % con alguna de las dos comorbilidades), lo que indica que la enfermedad en la población local se comporta de forma similar a lo reportado en el resto del mundo. Sin embargo, llama la atención que ninguno de nuestros pacientes refirió alergia alimentaria, otro de los componentes de la marcha atópica. Asimismo, se destaca que solo en el 7,1 % persistía el asma, es decir, la recuperación era del 84 % de los inicialmente asmáticos, lo que también concuerda con lo referido en la literatura especializada.

En cuanto a las condiciones neuropsiquiátricas en casos de dermatitis atópica, en varias revisiones sistemáticas se han encontrado asociaciones con el trastorno de déficit de atención con hiperactividad, con un OR entre 1,2 y 7,8 ^34^. Sin embargo, en un estudio en Medellín, se encontró que la frecuencia de este déficit en pacientes con dermatitis atópica fue similar a la de la población general; en tanto que, en el presente estudio, se encontró que el 38,1 % de los pacientes refería déficit de atención, una cifra superior a los reportes de frecuencia de la población general, incluso de la población local, en la que se han reportado valores del 15 a l16 %. Cabe resaltar, además, que el presente estudio se hizo en mayores de seis años y el promedio de edad de la mayoría era de 27 años, y los estudios locales se han hecho en pacientes menores, lo que indica que el déficit de atención ha persistido en los participantes en el estudio durante varios años, ya que el diagnóstico se hace cuando los síntomas se han iniciado antes de los 12 años. En cuanto a la depresión, la relación también es positiva y los OR reportados en un estudio de casos y controles fueron de 1,2 a 1,6 ^35^, aunque parece mejorar cuando los síntomas de la dermatitis atópica se hacen más leves.

En el presente estudio, se encontró que solo el 0,9 % reportó haber sufrido depresión, lo que quizá se deba a que, en general, los participantes presentaban una dermatitis atópica moderada, en tanto que la depresión suele asociarse con casos graves; en muchos casos se puede presentar ánimo disminuido, pero no de forma persistente, por lo que no se diagnostica el trastorno depresivo como tal. Asimismo, las alteraciones del sueño son muy frecuentes en pacientes con dermatitis atópica y afectan hasta al 60 % de ellos (33,36). En el presente estudio, se reportaron trastornos del sueño en 36,3 % de los participantes, lo que también concuerda con lo encontrado en un estudio previo en Medellín sobre enfermedades atópicas y trastornos neuropsiquiátricos, en el cual se encontró que, de todas las enfermedades atópicas evaluadas, la dermatitis atópica coexistía más frecuentemente con el insomnio, con un OR de 4,23 ^11^, lo que se explica por el intenso prurito que referían los pacientes, especialmente en las noches.

En cuanto a las comorbilidades metabólicas, la más relevante en los estudios de asociación ha sido la obesidad, con valores de OR entre 1,1 y 1,3 en las revisiones sistemáticas ^34^. En nuestro estudio, el 1,8 % de los pacientes presentaba obesidad, en tanto que las asociaciones han sido menos consistentes con otras condiciones metabólicas como el infarto de miocardio, el accidente cerebrovascular y la diabetes mellitus de tipo 2 ^35^. Localmente, tampoco se ha registrado una frecuencia significativa en casos de dermatitis atópica, lo que coincide con lo encontrado en el presente estudio, pues solo el 1,8 % de los pacientes era diabético y ninguno refirió condiciones cardiovasculares. Además, los pacientes con la enfermedad han reportado el inicio del tabaquismo a más temprana edad, mayor consumo de bebidas alcohólicas, menos actividad física y más frecuencia de dislipidemia ^32^, lo cual se correlaciona con lo hallado en el presente estudio, ya que 14,2 % de los pacientes reportaron tabaquismo activo y, casi la mitad (41,6 %), consumo de alcohol.

En lo referente a las enfermedades autoinmunitarias, también se han reportado asociaciones importantes con la enfermedad intestinal inflamatoria y la alopecia areata. Por último, en cuanto a las neoplasias, se ha observado asociación con el desarrollo de linfomas ^34^. En ese sentido, se interrogó a los pacientes sobre enfermedades autoinmunitarias y neoplasias y no se encontró ninguna en los pacientes evaluados; es posible que dichas enfermedades puedan aparecer en el transcurso de la vida de los individuos, por lo que se recalca la importancia del seguimiento y la tamización por sistemas.

En los estudios sobre el riesgo de dermatitis atópica, se ha descrito la relación entre tener un padre con atopia y la dermatitis atópica (OR reportados de 1,3), riesgo que aumenta a 2,08 cuando ambos padres la han padecido e, incluso, hasta 2,32 si presentan otros antecedentes atópicos además de la dermatitis ^31^^,^^36^^,^^37^. En nuestro estudio, se encontró que el antecedente familiar de primer grado con dermatitis atópica se presentaba en el 82,3 % de los casos, el asma en el 35,3 % y la rinitis alérgica en el 36,2 %, aunque no fue posible determinar en qué pacientes el antecedente era uniparental o biparental. Sin embargo, los datos sí corroboran la relevancia de la historia familiar como factor de riesgo de descendencia con dermatitis atópica.

En cuanto a los aspectos económicos, se sabe que la dermatitis atópica implica una carga económica elevada. En estudios en España sobre el impacto económico de la enfermedad, se ha descrito que esta implica un costo de hasta 2.400 euros anuales (entre 150 y 200 euros mensuales) por paciente, que incluso puede ascender a 4.545 euros cuando coexiste con otras enfermedades, especialmente el asma bronquial ^24^^,^^38^. En Colombia, no hay estudios sobre este aspecto y ,aunque el nuestro no pretendía evaluar el impacto económico, sí se encontró que la mayoría de los pacientes dependían económicamente de alguien más, casi la mitad de ellos no tenía ingresos fijos y la mitad invertía entre COP$ 100.000 y $ 500.000 mensuales para lidiar con su enfermedad, lo que refleja su impacto económico en una población que no cuenta con ingresos propios para acceder a las consultas y tratamientos, y depende de otros y de un sistema general de salud que no alcanza a cubrir todas las demandas de salud de la población.

Con respecto a la clasificación de la dermatitis atópica mediante las escalas EASI y SCORAD, en general, los pacientes analizados presentaban dermatitis atópica leve a moderada, con un porcentaje de superficie corporal afectado promedio del 17,14 %, que se considera moderado (entre 17 y 40 %). El puntaje en el EASI fue del 6,9, lo que representa un compromiso leve, aunque muy cercano al límite superior (leve: entre 1,1 y 7,0) ^39^, y se explicaría porque la gran mayoría de pacientes estaba en tratamiento y ello pudo reflejarse en una disminución de la gravedad de la enfermedad en el momento de la encuesta.

En el SCORAD, el puntaje fue de 32,4 (moderado: entre 25 y 50) ^26^^,^^39^, lo que se debería, de nuevo, al tratamiento que estaba recibiendo la gran mayoría de pacientes en el momento de la encuesta. Cabe resaltar una vez más que la edad promedio de los participantes fue de 27 años, es decir, pacientes con dermatitis atópica del adulto, que, según diversos estudios, tiene un comportamiento clínico más complicado y requiere la combinación de diversos tratamientos ^40^. Con respecto a la intensidad del prurito, en el estudio se reportó una intensidad promedio, casi de 6 en una escala análoga de 1 a 10, lo que concuerda con la gravedad de la enfermedad registrada en las otras escalas. Además, entre los estigmas de atopia más comunes se registraron la palidez centrofacial y el doble pliegue palpebral, este último uno de los más frecuentes en esta condición, aunque también la palidez es un hallazgo característico en los pacientes con dermatitis atópica ^41^^,^^42^.

En nuestra población de estudio la gran mayoría (92,9 %) había utilizado al menos uno de los tratamientos usuales, más frecuentemente el tópico. La fototerapia fue la otra estrategia de manejo más reportada (35,4 %), principalmente la UVB de banda estrecha (77,5 % de los tratados con fototerapia). La fototerapia es uno de los esquemas más utilizados con el tratamiento tópico, especialmente en pacientes con un grado leve de la enfermedad que no mejoran con el tratamiento inicial, o en aquellos con compromiso moderado. En cuanto al tratamiento sistémico, el medicamento más comúnmente utilizado fue el metotrexato (16,8 %), seguido por la ciclosporina (15,9 %), la azatioprina y los productos biológicos (cada uno con 13,3 %), y, por último, el micofenolato de mofetilo (10,6 %). En general, los estudios previos y la experiencia de otros centros señalan que la ciclosporina es el primer medicamento inmunosupresor utilizado, seguido por la azatioprina y, en tercer lugar, el metotrexato y el micofenolato ^43^. En cuanto al uso de productos biológicos, en el momento de la selección de los pacientes aún no se había autorizado en el país el dupilumab (anticuerpo monoclonal aprobado para el tratamiento de la dermatitis atópica) ^44^, y solo se empleaba el omalizumab en pacientes que también presentaban asma. Este medicamento está indicado para el tratamiento de la urticaria crónica y el asma, pero en la dermatitis atópica no ha demostrado un efecto palpable, aunque sí se ha reportado su gran utilidad en pacientes que también tienen asma, cuyos síntomas respiratorios mejoran ^45^.

En cuanto al impacto de la enfermedad en la calidad de vida, este se evaluó con el Skindex-29 y el EQ-5D, escalas usadas en enfermedades dermatológicas y ampliamente validadas. En general, se encontraron valores algo elevados en el Skindex-29, excepto para el dominio funcional, con un puntaje total de 24,7 (leve: 20-30); el dominio sintomático registró el valor más alto, con 37,7 (moderado: 30-40) y el emocional, con 25,09 (leve: 2030). En un estudio de la Asociación Colombiana de Dermatología y Cirugía Dermatológica (Asocolderma) publicado en el 2017 en el que se midió el impacto en la calidad de vida de varias enfermedades dermatológicas con el Skindex-29, se encontraron valores muy similares a los reportados en el presente estudio para la dermatitis atópica, con un puntaje global de 29,6, de 42,8 en el dominio sintomático, de 34,3 en el emocional y de 18 en el funcional ^28^, lo que denota el efecto de la enfermedad, sobre todo en el componente sintomático y, un poco menos, en el emocional.

La escala EQ-5D se empleó en 91 pacientes, ya que 21 de los participantes eran menores de edad y para ellos no se utilizó esta escala, y un adulto no contestó adecuadamente las preguntas. En los restantes (n=91), los síntomas más frecuentes fueron el dolor o el malestar (17,6 %) y la ansiedad o la depresión (12,1 %); asimismo, se encontró que el puntaje promedio para el estado de salud reportado por los participantes el día de la evaluación fue de 75,01. Se esperaría que, en pacientes con dermatitis atópica, el dolor o malestar y la ansiedad o depresión fueran los componentes más relevantes, dado que uno de los síntomas más importantes de la enfermedad es el prurito y este se asocia con síntomas psiquiátricos como ansiedad, irritabilidad, alteraciones del sueño, tristeza, minusvalía y, en general, ánimo disminuido, también reportados en nuestro estudio, y que explicarían el promedio de 75,01 para la percepción del estado de salud. También, debe señalarse que, aunque la frecuencia de depresión fue solo de 0,9 %, sí hubo más pacientes que identificaron los síntomas depresivos en esta escala, lo que sugiere que la frecuencia de la depresión podría ser mayor, o que existe el riesgo de desarrollarla en el futuro.

Entre las limitaciones del estudio, debe mencionarse la ausencia de pacientes menores de seis años, puesto que se trata de un grupo poblacional bastante afectado por la enfermedad, aunque cabe resaltar la importancia de la población adulta como grupo afectado. Otra limitación fue la falta de algunos datos que serían relevantes, como la frecuencia de los antecedentes familiares uniparentales o biparentales, lo que hubiera podido fortalecer el poder estadístico. Sin embargo, con los datos disponibles fue posible observar el efecto en la calidad de vida. En cuanto a las escalas de evaluación, se sabe que hay otras herramientas utilizadas extensamente en la práctica clínica, como el *Dermatology Life Quality Index* (DLQI), instrumento que, a diferencia del Skindex-29 y el EQ5D, aún no se validado en Colombia. Asimismo, dado que no existen escalas validadas para evaluar el impacto de esta enfermedad en la calidad de vida de los menores de 16 años, fue imposible hacer un adecuado análisis de este grupo tan importante de pacientes; sin embargo, como ya se mencionó, solo el 20 % de la población de estudio correspondía a menores de edad, por lo que estos datos faltantes no cambiarían los resultados.

La fortaleza del estudio radica en la inclusión de un importante número de pacientes, lo que permitió aproximarse a una caracterización regional de esta enfermedad, útil para comparaciones futuras con otras áreas de Colombia y del mundo.

El estudio aporta al conocimiento sobre dermatitis atópica en el ámbito nacional. Los resultados concuerdan con lo reportado internacionalmente e, incluso, con los pocos datos locales disponibles. Se destaca la presentación de la enfermedad en la edad adulta y el promedio de gravedad de leve a moderada, con un impacto especialmente en lo sintomático y emocional (componente neuropsiquiátrico), así como la frecuencia del trastorno de déficit de atención con hiperactividad como comorbilidad, los trastornos del sueño y los síntomas depresivos. Se resalta, además, la presentación de otras formas de atopia, especialmente el asma, aunque esta parece mejorar con la edad.
